# Experimental Study on the Strength Characteristics and Water Permeability of Hybrid Steel Fibre Reinforced Concrete

**DOI:** 10.1155/2014/518640

**Published:** 2014-10-29

**Authors:** M. P. Singh, S. P. Singh, A. P. Singh

**Affiliations:** Dr. B. R. Ambedkar National Institute of Technology, Jalandhar 144 011, India

## Abstract

Results of an investigation conducted to study the effect of fibre hybridization on the strength characteristics such as compressive strength, split tensile strength, and water permeability of steel fibre reinforced concrete (SFRC) are presented. Steel fibres of different lengths, that is, 12.5 mm, 25 mm, and 50 mm, having constant diameter of 0.6 mm, were systematically combined in different mix proportions to obtain mono, binary, and ternary combinations at each of 0.5%, 1.0%, and 1.5% fibre volume fraction. A concrete mix containing no fibres was also cast for reference purpose. A total number of 1440 cube specimens of size 100∗100∗100 mm were tested, 480 each for compressive strength, split tensile strength, and water permeability at 7, 28, 90, and 120 days of curing. It has been observed from the results of this investigation that a fibre combination of 33% 12.5 mm + 33% 25 mm + 33% 50 mm long fibres can be adjudged as the most appropriate combination to be employed in hybrid steel fibre reinforced concrete (HySFRC) for optimum performance in terms of compressive strength, split tensile strength and water permeability requirements taken together.

## 1. Introduction

Concrete is one of the most widely used construction materials in the world. Its properties like easy mould ability into any desired shape, easy availability of its constituent materials, cost effectiveness, and many other advantages make it popular construction material. However, concrete does have few deficiencies like brittleness, low tensile strength, and low ductility. The tensile strength of concrete is in the range of 7–10% of its compressive strength.

Durability is the basic requirement of any concrete structure as it should be able to withstand all stresses and remain functional throughout its designed life span. Some of the reasons for failure of structures are poor design, use of poor quality materials, bad workmanship, deterioration of concrete due to ingress of harmful ingredients, and so forth. Water is generally involved in any form of deterioration, and, in porous solids, permeability of the material to water usually determines the rate of deterioration.

Thus, of the many factors affecting the durability of concrete structures, permeability has been identified as one of the key factors [1]. Permeability of concrete is defined as the ease with which a fluid, liquid, or gas flows through it under pressure. The lower the permeability of concrete would be, the more durable the concrete would be.

Many factors like cement content, cement type, aggregate type, shape and size, admixtures in cement, super plasticizers, curing age, curing temperature, methods of curing, and so forth have been found to affect the permeability of concrete [2, 3]. Many investigators have carried out studies on the effect of the above-mentioned factors on the permeability of concrete. The effects of pozzolanic materials like silica fume, fly ash, and rice husk ash on permeability of concrete have also been investigated [4–7].

Fibre reinforced concrete (FRC) is a composite material comprising cement, aggregate, and randomly distributed discrete fibres. Normal unreinforced concrete is brittle with a low tensile strength and strain capacity. Short, discrete, and randomly distributed fibres have been found to be very effective in overcoming some of the deficiencies of conventional concrete. A lot of research has been carried out on steel fibre reinforced concrete (SFRC) using mono steel fibres and it has been observed that the addition of fibres improves its compressive strength up to some extent and considerably increases the tensile strength, flexural strength, shear strength, and flexural toughness [8–16]. The degree of improvement in the above properties depends on many factors including size, type, shape, aspect ratio, and volume fraction of fibres used [17–19].

In the last decade, attempts have been made to study the effect of addition of fibres on permeability of FRC using mono fibres. Researchers have used variety of mono fibres like steel, carbon, polypropylene, polyvinyl alcohol, nylon, and glass to study the permeability characteristics of cracked and uncracked concrete [20–26].

Mainly the fibres are divided into two types, that is, metallic and nonmetallic. Steel and carbon fibres are termed as metallic fibres and fibres like polymeric, carbon, glass, and naturally occurring fibres are clubbed under the umbrella of nonmetallic fibres [27–32]. Further, depending upon the length of the fibre, these are classified as macro- and microfibres. Normally, researchers have been using only mono fibres in their studies. The concept of hybridization of fibres came into light in the last decade. Hybridization of fibres means incorporating fibres of different materials or fibres of the same material having different lengths/aspect ratios [33–38]. Hybrid steel fibre reinforced concrete (HySFRC) is the most recent advancement in the field of FRC. With the increase in the structural and mechanical properties of concrete, it is imperative to increase the durability of these materials.

To open new application areas, HySFRC should be designed to perform adequately in terms of workability, strength, ductility, and, most importantly, the durability. Utilizing the concept of hybridization, concrete with superior properties can be developed. The durability of concrete depends mainly upon its resistance to ingress of moisture. The moisture which enters into the concrete can lead to corrosion of steel reinforcement and considerably reduces the life of the structures. As such, the durability of concrete depends largely upon the permeability of concrete which is defined as the ease with which it allows the fluids to pass through it. Even though durability is a key factor affecting longevity of concrete structures, only limited studies were carried out to investigate the effect of addition of different steel fibres on the durability of concrete.

## 2. Research Significance

A comprehensive review of literature, which was carried out but reported briefly in the preceding paragraphs, indicates that the effect of addition of hybrid steel fibres on the permeability and strength characteristic of HySFRC has not been investigated so far and the information on the subject is scanty. In this study, therefore, an attempt has been made to investigate the combined effect of addition of hybrid steel fibres on the water permeability and strength characteristics such as compressive strength and split tensile strength of HySFRC mixes made with different fibre volume fractions, each volume fraction containing different combinations of steel fibres of varying lengths.

## 3. Experimental Procedure

### 3.1. Materials and Mix-Proportioning

The proportion of the ingredients by weight constituting the reference concrete mix was 1 : 1.52 : 1.88 with a water-cement ratio of 0.46 by weight. The 28-day compressive strength of the reference concrete mix was 38.65 MPa. Pozzolanic Portland cement, crushed stone coarse aggregates having maximum size of 12 mm, and locally available river sand were used. The materials conformed to the relevant Indian standard specifications [39, 40]. Corrugated steel fibres, 12.5 mm, 25 mm, and 50 mm long, each with constant diameter of 0.6 mm, were used in different combinations by weight. Three volume fractions of the fibres, that is, 0.5%, 1.0%, and 1.5%, were used, each volume fraction containing different mix proportions of steel fibres of different aspect ratios in mono, binary, and ternary combinations.  Table 1 presents 13 such fibre mix combinations corresponding to each fibre volume fraction of 0.5%, 1.0%, and 1.5% resulting in a total of 39 fibre concrete mixes. In addition, concrete containing no fibres was also cast for reference purpose. In all 40 concrete mixes were prepared in this investigation.

### 3.2. Workability of SFRC and HySFRC and Casting of Specimens

The inverted slump cone test was used to measure the workability of concrete containing different combinations of steel fibres. This test has been specifically developed to measure workability of FRC. The time taken to empty the cone for all the mixes used in this investigation was within limits prescribed for FRC except for some mixes made with 1.5% volume fraction of fibres where it was rather difficult to maintain the workability within limits even with increased dose of superplasticizer and fibre balling was observed in those mixes during mixing.

The casting of the specimens was done under laboratory conditions using standard equipment. Each mix of concrete consisted of standard cube specimens of 100∗100∗100 mm size for compressive strength, split tensile strength, and water permeability tests which were conducted after 7, 28, 90, and 120 days of curing in potable water. For all the 40 concrete mixes, a total number of 1440 cube specimens, 480 specimens each for compressive strength, split tensile strength, and water permeability, were cast.

### 3.3. Compressive Strength Tests

Compressive strength tests were conducted in accordance with IS: 516–1956 on a 2000 kN compression testing machine. The bearing surfaces of the machine were cleaned and the test specimen was placed in the machine such that the load was applied to the faces other than the cast face of the specimen. The maximum compressive load on the specimen was recorded as the load at which the specimen failed to take further increase in the load. The average of three specimens was taken as the representative value of compressive strength for each mix. The compressive strength was calculated by dividing the maximum compressive load by the cross-sectional area of the cube specimen over which the load was applied.

### 3.4. Split Tensile Strength Tests

The split tensile strength was conducted on the compression testing machine by placing the specimen diagonally. The procedure adopted in this investigation for split tensile strength tests was the same as used in some previous investigations [41, 42]. The split tensile strength was determined by using the following formula:

(1) $$\begin{array}{c} \sigma \text{spt}=\frac{0.544P}{a^{2}}, \end{array}$$

where *σ*spt is split tensile strength in MPa, *P* is splitting load in N, and *a* is size of cube specimen in mm.

### 3.5. Water Permeability Tests

Water permeability of fibrous concrete was obtained by testing specimens in a water permeability tester. The water permeability was conducted as per the procedure laid out in IS: 3085–1965 [43]. The test cells of the permeability testing equipment have a cross section of 115 mm ∗ 115 mm. The annular space between the test mould and the cube was filled with a mix of resin and wax mixed in the ratio of 2 : 1 by volume. Prior to testing, the specimens were surface-dried and painted with a mixture of resin and wax, on all the faces except the two faces through which unidirectional flow of water under requisite pressure was planned to take place. The annular space in the bottom portion of the test mould was tightly packed with jute pieces soaked in the molten mixture. This mixture in its smoking state was added to fill the remaining portion of the annular space and was compressed by a steel ruler to release any entrapped air. The seal was allowed to harden for 24 hours and then was checked by passing air from the bottom while covering the top surface with the layer of water. Absence of any air bubble emerging out of the seal confirmed that the seal was perfect. The sealing ensured a unidirectional flow from top to bottom through the sample and not from sides. The test cell was designed to withstand a working pressure of 1.5 MPa and the specimens in the present study were tested at pressure ranging from 0.8 MPa to 1.0 MPa. The discharge measurements were taken at regular intervals till the attainment of steady state which was confirmed when the discharge passing through the specimen becomes constant. After attaining the steady state, readings were taken at regular intervals for 48 hours. The average discharge was used for determining the coefficient of permeability. The steady state was normally attained within a test period of 7 days to 15 days. The coefficient of permeability was calculated by using the following formula:

(2) $$\begin{array}{c} k=\frac{QL}{AH}, \end{array}$$

where *k* is coefficient of permeability, m/s, *Q* is rate of discharge, cumecs, *L* is dimension of the specimen measured in the direction of flow, *A* is area of cross section of the specimen, and *H* is water head causing flow measured, m.

## 4. Results and Discussion

In all, 39 mixes, 13 each for volume fraction of 0.5%, 1.0%, and 1.5% containing different combinations of steel fibres, were tested for compressive strength, split tensile strength, and water permeability at 7, 28, 90, and 120 days of curing. A mix containing no steel fibres was also tested as control/reference mix. Results for different mixes containing different combinations of steel fibres at 28 days of curing are presented in the following sections as the trends are more or less similar at other curing ages.

### 4.1. Compressive Strength

#### 4.1.1. Mono Steel Fibre Reinforced Concrete

**Figure 1:**
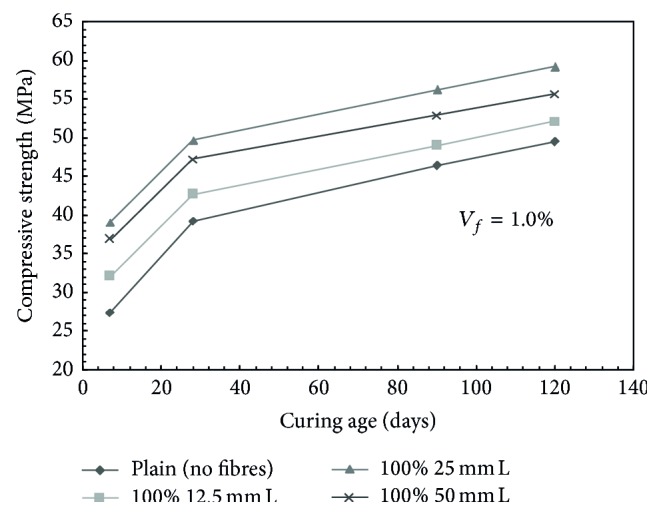
Compressive strength versus age of curing for mono steel fibres mixes; *V*_*f*_ = 1.0%; L: long fibers.

For mono steel fibre mixes, at a volume fraction of 0.5%, the compressive strength was found to increase by 5.28%, 24.06%, and 16.82% with the addition of 100% 12.5 mm, 100% 25 mm, and 100% 50 mm long fibres, respectively, over plain concrete mix at 28 days of curing. Similarly, for a mix containing 1.0% volume fraction, the compressive strength was found to increase by 8.85%, 26.61%, and 20.32% with the addition of 100% 12.5 mm, 100% 25 mm, and 100% 50 mm long fibres, respectively, over plain concrete mix at 28 days of curing. For 1.5% volume fraction, increase in the compressive strength of the order of 13.69%, 29.54%, and 14.45% was observed with the addition of 100% 12.5 mm, 100% 25 mm, and 100% 50 mm long fibres, respectively, over plain concrete.  Figure 1 presents typical trends for compressive strength of mixes containing mono steel fibres at a fibre volume fraction of 1.0%.

**Figure 2:**
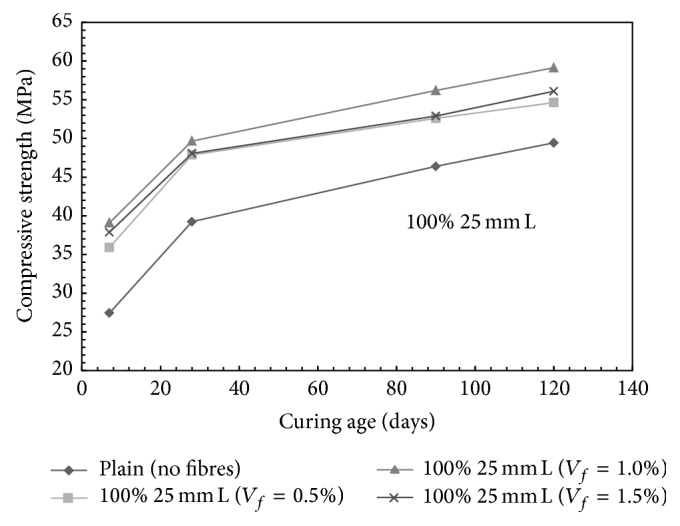
Compressive strength versus age of curing for mono steel fibres mixes (100% 25 mm long fibres); L: long fibers.

For mono steel fibre mixes, with the addition of 100% 12.5 mm long fibres, the compressive strength was increased by 5.28%, 8.85%, and 13.69% for 0.5%, 1.0%, and 1.5% fibre content, respectively, at 28 days of curing. Similarly, with the addition of 100% 25 mm long fibres, the compressive strength was increased by 24.06%, 26.61%, and 29.54% for 0.5%, 1.0%, and 1.5% fibre content, respectively, at 28 days of curing. For 100% 50 mm long fibres, an increase in the compressive strength of the order of 16.82%, 20.32%, and 14.45% was observed for 0.5%, 1.0%, and 1.5% fibre content, respectively. The drop in compressive strength at 1.5% volume fraction for a mix with 100% 50 mm long fibres may be attributed to the balling effect of the steel fibres during mixing.  Figure 2 presents the influence of fibre volume fraction on the compressive strength of mono steel fibre mixes made with 100% 25 mm long fibres.

The optimum fibre length for compressive strength for all the volume fractions tested for SFRC mixes is 25 mm, whereas 12.5 mm long fibres performed poorly. This may be due to the fact that some minimum fibre length is required to resist the cracks and their propagation. For 50 mm long fibres, their number for particular volume fraction is less as compared to 25 mm fibres and thus may not be as effective in arresting the propagation of cracks, thus giving poor performance compared to 25 mm fibres.

#### 4.1.2. Effect of Fibre Hybridization

First, the results of HySFRC mixes containing binary combinations of steel fibres are discussed and the results of HySFRC mixes containing ternary combinations of steel fibres are presented later.

**Figure 3:**
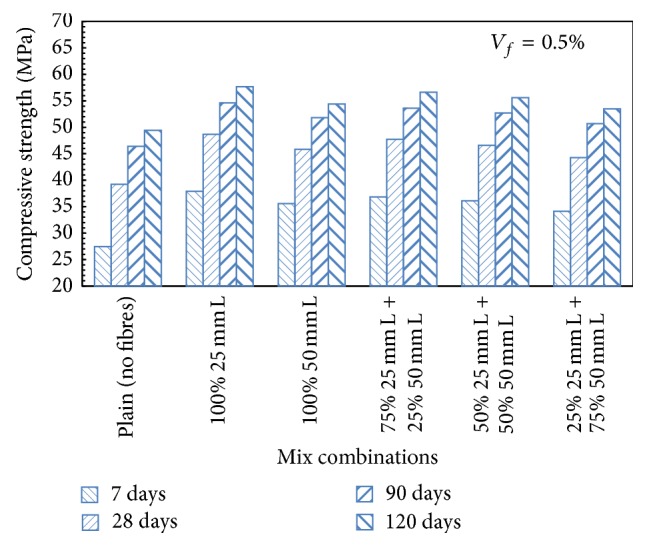
Compressive strength for HySFRC binary mixes; *V*_*f*_ = 0.5%; L: long fibers.

It has been observed that, with the addition of 75% 12.5 mm + 25% 25 mm long steel fibres presented in Figure 5, increase in the compressive strength of the order of 8.82%, 11.06%, and 10.88% for 0.5%, 1.0%, and 1.5% fibre content, respectively, over plain concrete mix was observed at 28 days of curing. For HySFRC mix which contained 50% 12.5 mm + 50% 25 mm long steel fibres, the compressive strength was increased by 11.65%, 13.51%, and 10.55% for 0.5%, 1.0%, and 1.5% volume fraction, respectively, over plain concrete mix at 28 days of curing.  Figure 3 presents a typical trend for HySFRC mixes containing binary combinations of 25 mm and 50 mm long steel fibres for a fibre volume fraction of 0.5%.

Similar trends were observed for a mix containing 25% 12.5 mm + 75% 25 mm, and the increase in the compressive strength over plain concrete mix was 14.96%, 17.26%, and 15.47% for 0.5%, 1.0%, and 1.5% fibre content, respectively, at 28 days of curing. Similarly, for binary steel fibre mixes of 100% 12.5 mm and 100% 50 mm long steel fibres, with the addition of 75% 12.5 mm + 25% 50 mm long steel fibres, increase in the compressive strength of the order of 15.73%, 12.67%, and 10.55% for 0.5%, 1.0%, and 1.5% fibre content, respectively, over plain concrete mix was observed at 28 days of curing. For HySFRC mix, which contained 50% 12.5 mm + 50% 50 mm long steel fibres, the compressive strength was increased by 10.88%, 14.89%, and 12.41% for 0.5%, 1.0%, and 1.5% volume fraction, respectively, over plain concrete mix at 28 days of curing. Similar trends were observed for a mix containing 25% 12.5 mm + 75% 50 mm, and the increase in the compressive strength over plain concrete mix was 14.22%, 17.51%, and 14.84% for 0.5%, 1.0%, and 1.5% fibre content, respectively.

In the binary mix combinations of 100% 25 mm and 100% 50 mm long steel fibres, with the addition of 75% 25 mm + 25% 50 mm long steel fibres, increase in the compressive strength of the order of 21.59%, 21.72%, and 13.87% for 0.5%, 1.0%, and 1.5% fibre content, respectively, over plain concrete mix was observed at 28 days of curing. For HySFRC mix, which contained 50% 25 mm + 50% 50 mm long steel fibres, the compressive strength was increased by 18.79%, 23.88%, and 21.34% for 0.5%, 1.0%, and 1.5% volume fraction, respectively, over plain concrete mix at 28 days of curing. Similar trends were observed for a mix containing 25% 25 mm + 75% 50 mm, and the increase in the compressive strength over plain concrete mix was 12.82%, 22.18%, and 16.67% for 0.5%, 1.0%, and 1.5% fibre content, respectively.

**Figure 4:**
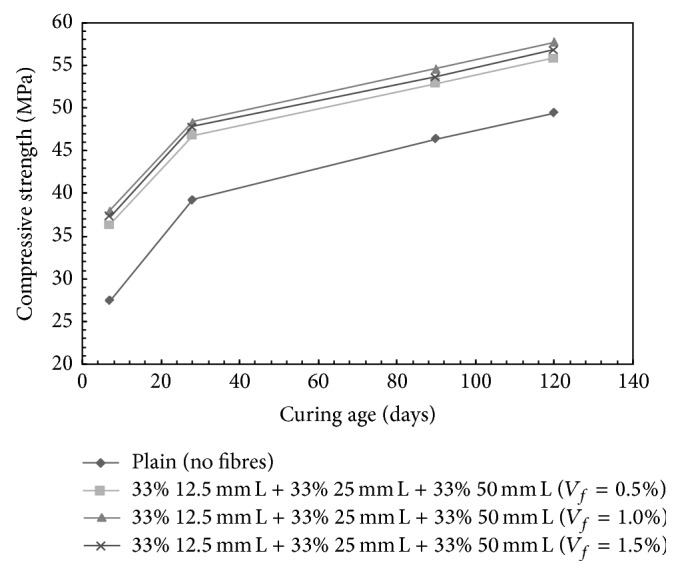
Compressive strength for HySFRC ternary mixes; L: long fibers.

However, for the ternary mix combination containing 33% 12.5 mm + 33% 25 mm + 33% 50 mm long steel fibres, results are presented in Figure 4; the increase in the compressive strength over plain concrete was observed to be 19.35%, 23.37%, and 21.92% for 0.5%, 1.0%, and 1.5% volume fraction, respectively, at 28 days of curing.

For a particular fibre volume fraction, the highest compressive strength is obtained for a mix made with 100% 25 mm steel fibres. Similarly, for a particular fibre volume fraction, the lowest value of the compressive strength is given by a mix made with 100% 12.5 mm long fibre followed by a mix made of 75% 12.5 mm + 25% 25 mm long fibres. As the amount of short fibres in a particular mix is increased or short fibres are partially or fully replaced by relatively long fibres, a decrease in the compressive strength is observed. Further, at all the fibre mixes tested in this investigation, the lowest compressive strength is obtained for a mix made with 100% 12.5 mm long fibres at a fibre volume fraction of 0.5%, whereas the highest compressive strength is given by a mix made of 100% 25 mm long fibres at a fibre volume fraction of 1.5%.

It may also be noted that the compressive strength increase is more sensitive to fibre length than volume fraction in case of binary combinations of fibre. However, in case of ternary combinations, the influence of fibre volume fraction is almost negligible.

### 4.2. Split Tensile Strength

#### 4.2.1. Mono Steel Fibre Reinforced Concrete

**Figure 5:**
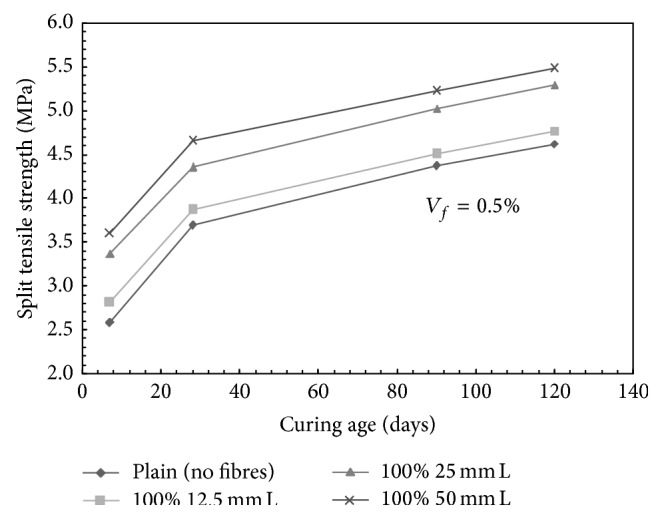
Split tensile strength versus age of curing for mono steel fibres mixes; *V*_*f*_ = 0.5%; L: long fibers.

For mono steel fibre mixes, at a volume fraction of 0.5%, the split tensile strength was found to increase by 4.88%, 18.16%, and 26.29% with the addition of 100% 12.5 mm, 100% 25 mm, and 100% 50 mm long fibres, respectively, over plain concrete mix at curing of 28 days. Similarly, for a mix containing 1.0% volume fraction of fibres, the split tensile strength was found to increase by 10.57%, 24.66%, and 37.40% with the addition of 100% 12.5 mm, 100% 25 mm, and 100% 50 mm long fibres, respectively, over plain concrete mix at 28 days of curing. For 1.5% volume fraction, increase in the split tensile strength of the order of 12.74%, 31.98%, and 14.09% was observed with the addition of 100% 12.5 mm, 100% 25 mm, and 100% 50 mm long fibres, respectively, over plain concrete. Figure 5 presents typical trends for split tensile strength of mixes containing mono steel fibres at a fibre volume fraction of 0.5%.

For mono steel fibre mixes, for mixes made with 100% 12.5 mm long fibres, the split tensile strength was increased by 4.88%, 10.57%, and 12.74% for 0.5%, 1.0%, and 1.5% fibre content, respectively, at 28 days of curing. Similarly, for mixes containing 100% 25 mm long fibres, the split tensile strength was increased by 18.16%, 24.66%, and 31.98% for 0.5%, 1.0%, and 1.5% fibre content, respectively, at 28 days of curing. For mixes with 100% 50 mm long fibres, an increase in the split tensile strength of the order of 26.29%, 37.40%, and 14.09% was observed for 0.5%, 1.0%, and 1.5% fibre content, respectively.

**Figure 6:**
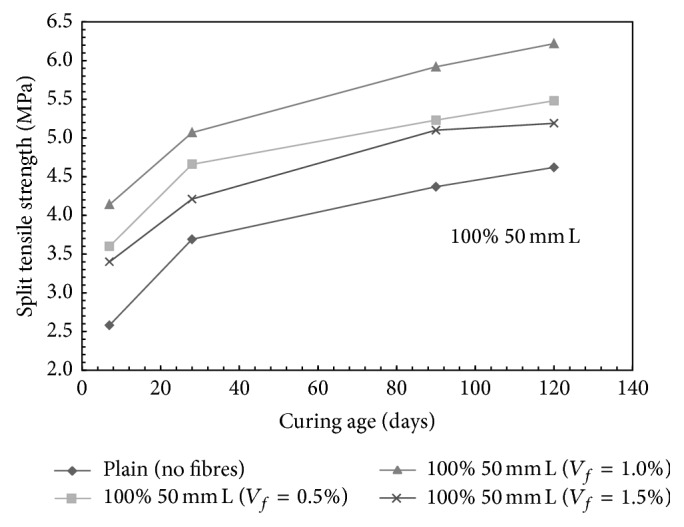
Split tensile strength versus age of curing for mono steel fibres mixes (100% 50 mm long fibres); L: long fibers.

It was observed that the difference in the split tensile strength for all the mixes at fibre contents of 1.0% and 1.5% was not significant except for 100% 50 mm long fibres, which may be attributed to the balling effect of the steel fibres during mixing at 1.5% volume fraction.  Figure 6 presents the influence of fibre volume fraction on the split tensile strength of mono steel fibre mixes made with 100% 50 mm long fibres.

#### 4.2.2. Effect of Fibre Hybridization

Like compressive strength results, the split tensile strength results of HySFRC mixes containing binary combinations of steel fibres are discussed first and the results of HySFRC mixes containing ternary combinations of steel fibres are presented later.

It has been observed that, with the addition of 75% 12.5 mm + 25% 25 mm long steel fibres, increase in the split tensile strength of the order of 7.05%, 13.82%, and 14.36% for 0.5%, 1.0%, and 1.5% fibre volume fraction, respectively, over plain concrete mix was observed at 28 days of curing. For HySFRC mix, which contained 50% 12.5 mm + 50% 25 mm long steel fibres, the split tensile strength was increased by 10.84%, 17.07%, and 15.72% for 0.5%, 1.0%, and 1.5% volume fraction, respectively, over plain concrete mix at 28 days of curing. Similar trends were observed for a mix containing 25% 12.5 mm + 75% 25 mm and the increase in the split tensile strength over plain concrete mix was 12.47%, 20.87%, and 19.24% for 0.5%, 1.0%, and 1.5% fibre content, respectively, at 28 days of curing.

Similarly, for binary steel fibre mixes of 100% 12.5 mm and 100% 50 mm long steel fibres, with the addition of 75% 12.5 mm + 25% 50 mm long steel fibres, increase in the split tensile strength of the order of 8.67%, 18.70%, and 21.68% for 0.5%, 1.0%, and 1.5% fibre content, respectively, over plain concrete mix was observed at 28 days of curing. For HySFRC mix, which contained 50% 12.5 mm + 50% 50 mm long steel fibres, the split tensile strength was increased by 15.72%, 22.76%, and 17.34% for 0.5%, 1.0%, and 1.5% volume fraction, respectively, over plain concrete mix at 28 days of curing. Similar trends were observed for a mix containing 25% 12.5 mm + 75% 50 mm, and the increase in the split tensile strength over plain concrete mix was 19.78%, 32.25%, and 22.76% for 0.5%, 1.0%, and 1.5% fibre content, respectively.

**Figure 7:**
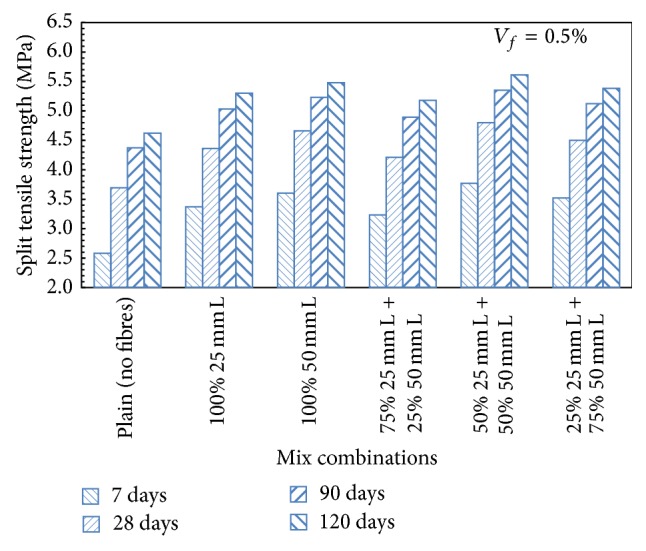
Split tensile strength for HySFRC binary mixes; *V*_*f*_ = 0.5%; L: long fibers.

In the binary mix combinations of 100% 25 mm and 100% 50 mm long steel fibres, with the addition of 75% 25 mm + 25% 50 mm long steel fibres, increase in the split tensile strength of the order of 14.09%, 27.37%, and 17.89% for 0.5%, 1.0%, and 1.5% fibre content, respectively, over plain concrete mix was observed at 28 days of curing. For HySFRC mix which contained 50% 25 mm + 50% 50 mm long steel fibres, the split tensile strength was increased by 30.08%, 33.60%, and 31.71% for 0.5%, 1.0%, and 1.5% volume fraction, respectively, over plain concrete mix at 28 days of curing. Similar trends were observed for a mix containing 25% 25 mm + 75% 50 mm, and the increase in the split tensile strength over plain concrete mix was 21.95%, 30.62%, and 27.10% for 0.5%, 1.0%, and 1.5% fibre content, respectively, at 28 days of curing.  Figure 7 presents a typical trend for HySFRC mixes containing binary combinations of 25 mm and 50 mm long steel fibres for a fibre volume fraction of 0.5%.

**Figure 8:**
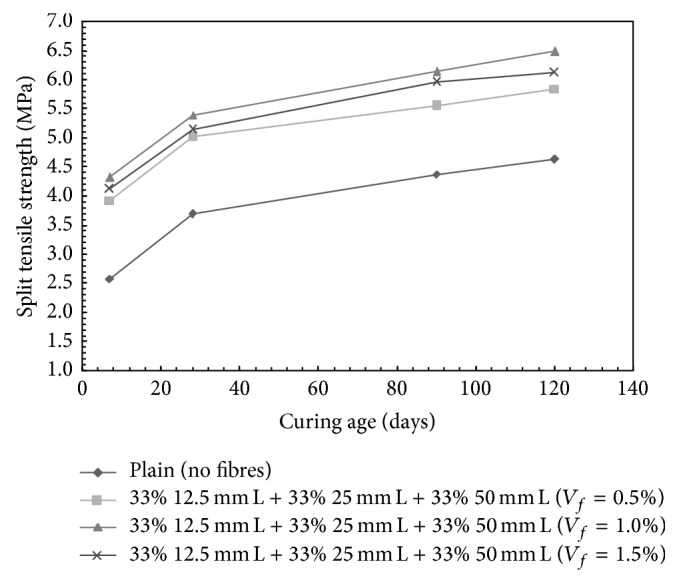
Split tensile strength for HySFRC ternary mixes; L: long fibers.

However, for HySFRC ternary mix containing 33% 12.5 mm + 33% 25 mm + 33% 50 mm long steel fibres for which the results are presented in Figure 8, the increase in the split tensile strength over plain concrete was observed to be 35.77%, 46.07%, and 39.30% for 0.5%, 1.0%, and 1.5% volume fraction, respectively, at 28 days of curing.

For a particular fibre volume fraction, the highest split tensile strength is obtained for a mix made with 33% 12.5 mm + 33% 25 mm + 33% 50 mm long steel fibres followed by a mix made with 50% 25 mm + 50% 50 mm long fibres. Similarly, for a particular fibre volume fraction, the lowest value of the split tensile strength is given by a mix made with 100% 12.5 mm long fibre followed by a mix made of 75% 12.5 mm + 25% 25 mm long fibres. Further, at all the fibre mixes tested in this investigation, the lowest split tensile strength is obtained for a mix made with 100% 12.5 mm long fibres at a fibre volume fraction of 0.5%, whereas the highest split tensile strength is given by a mix made of 33% 12.5 mm + 33% 25 mm + 33% 50 mm long steel fibres at a fibre volume fraction of 1.0%.

It may also be noted that the split tensile strength increase is more sensitive to fibre length than volume fraction in case of binary combinations of fibre. However, in case of ternary combinations, the influence of fibre volume fraction is almost negligible.

### 4.3. Water Permeability

#### 4.3.1. Mono Steel Fibre Reinforced Concrete

**Figure 9:**
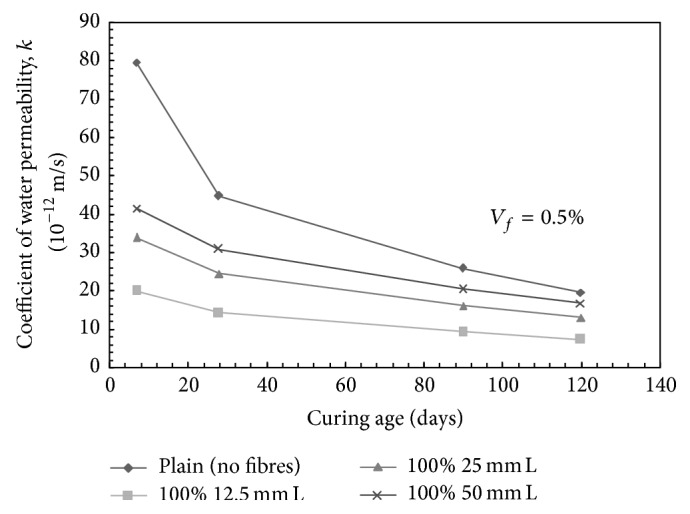
Coefficient of water permeability versus age of curing for mono steel fibres mixes; *V*_*f*_ = 0.5%; L: long fibers.

For mono steel fibre reinforced concrete (SFRC) mixes, for volume fraction of 0.5% and at 28 days of curing, the coefficient of water permeability was found to decrease over plain concrete mix (control mix) by 68.42%, 45.45%, and 31.33% with the addition of 100% 12.5 mm, 100% 25 mm, and 100% 50 mm long fibres, respectively. Similarly, for a mix containing 1.0% volume fraction of steel fibres and at 28 days of curing, the coefficient of water permeability was found to decrease over plain concrete mix by 79.21%, 65.23%, and 51.94% with the addition of 100% 12.5 mm, 100% 25 mm, and 100% 50 mm long fibres, respectively. For 1.5% volume fraction, decrease in the coefficient of water permeability of the order of 45.94%, 25%, and −2.61% at 28 days of curing was observed with the addition of 100% 12.5 mm, 100% 25 mm, and 100% 50 mm long fibres, respectively, over plain concrete. The increase in coefficient of water permeability at 1.5% volume fraction may be attributed to the balling effect of the steel fibres during mixing.  Figure 9 presents the variation of coefficient of water permeability with curing period for different mixes containing mono steel fibres for *V*_*f*_ = 0.5%.

The decrease in the water permeability of SFRC mixes may be attributed to the fact that a considerable reduction is reported in the occurrence of shrinkage cracks with the addition of fibres. Plastic shrinkage cracks occur during 24 hours of casting. The reduction in the shrinkage cracks helps in reducing the water permeability of concrete. Also, the drying shrinkage cracks get reduced to the extent of 25% up to an age of 28 days with the addition of steel fibres. Further, addition of impermeable steel fibres helps in breaking the continuity or interconnectivity of porous channels present in the concrete, thus resulting in lower water permeability. Also the maximum decrease in the water permeability of SFRC mixes made with 12.5 mm long fibres as compared to relatively long fibres such as 25 mm and 50 mm may be attributed to the fact that, for particular fibre volume fraction, the short fibres are more in number and are more effective in breaking the paths through which water can travel in concrete.

#### 4.3.2. Effect of Fibre Hybridization

First, the results of HySFRC mixes containing binary combinations of steel fibres are discussed and the results of HySFRC mixes containing ternary combinations of steel fibres are presented later.

**Figure 10:**
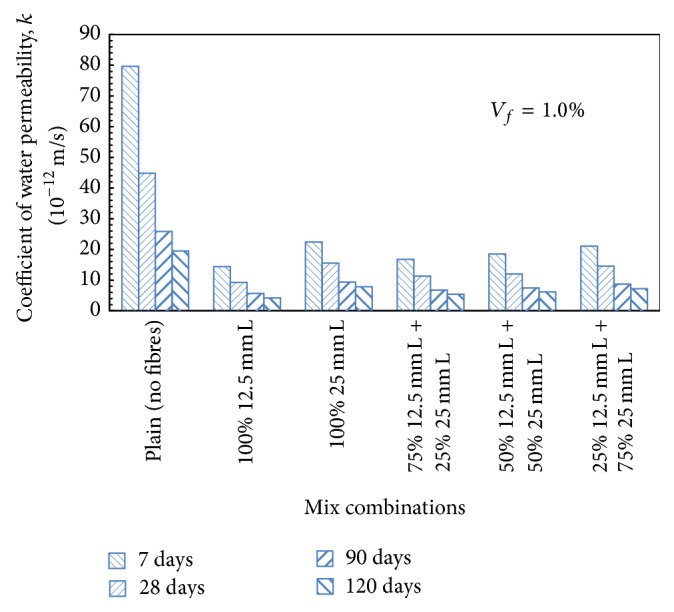
Coefficient of water permeability for HySFRC binary mixes; *V*_*f*_ = 1.0%; L: long fibers.

It has been observed that, with the addition of 75% 12.5 mm + 25% 25 mm long steel fibres, decrease in the coefficient of water permeability of the order of 66.57%, 74.75%, and 42.46% for 0.5%, 1.0%, and 1.5% fibre content, respectively, over plain concrete mix was observed after 28 days of curing. Similarly, for HySFRC mix, which contained 50% 12.5 mm + 50% 25 mm long steel fibres, the coefficient of water permeability was decreased by 59.79%, 73.08%, and 50.80% for 0.5%, 1.0%, and 1.5% volume fraction, respectively, over plain concrete mix after 28 days of curing. Similar trends were observed for a mix containing 25% 12.5 mm + 75% 25 mm, and the decrease in the coefficient of water permeability over plain concrete mix was 55.46%, 67.44%, and 29.66% for 0.5%, 1.0%, and 1.5% fibre content, respectively.  Figure 10 presents typical trends for HySFRC mixes containing binary combinations of 12.5 mm and 25 mm long steel fibres for a fibre volume fraction of 1.0%.

For binary mixes made with 12.5 mm and 50 mm long steel fibres and 25 mm and 50 mm long steel fibres, similar trends were observed and the detailed results are not presented here as the same are submitted to another journal.

**Figure 11:**
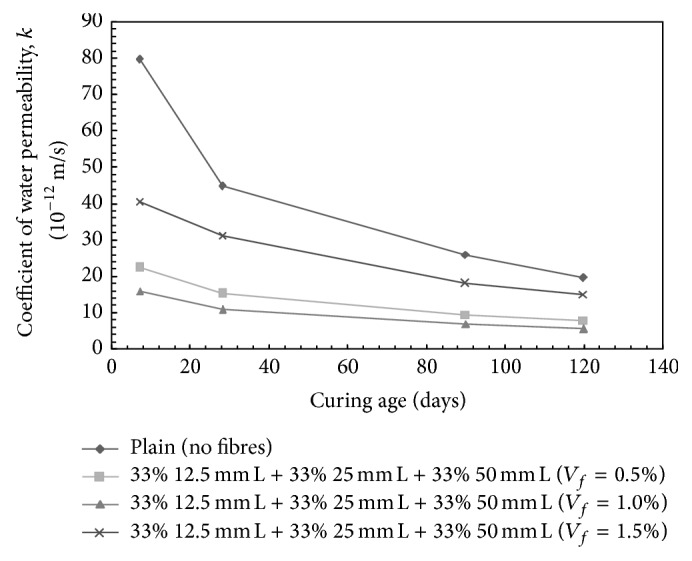
Coefficient of water permeability for HySFRC ternary mixes; L: long fibers.

However, for the ternary mix combination containing 33% 12.5 mm + 33% 25 mm + 33% 50 mm long steel fibres (Figure 11), the decrease in the coefficient of water permeability over plain concrete was observed to be 65.99%, 75.74%, and 30.46% for 0.5%, 1.0%, and 1.5% volume fraction, respectively.

For a particular fibre volume fraction, the lowest values of the coefficient of water permeability are obtained for a mix made with 100% 12.5 mm steel fibres followed by a mix made of 33% 12.5 mm + 33% 25 mm + 33% 50 mm long fibres. Similarly, for a particular fibre volume fraction, the highest value of the water permeability is given by a mix made with 100% 50 mm long fibre followed by a mix made of 25% 12.5 mm + 75% 50 mm long fibres.

As the amount of short fibres in a particular mix is decreased or short fibres are partially or fully replaced by relatively long fibres, an increase in the water permeability is observed. Further, at all the fibre mixes tested in this investigation, the lowest coefficient of water permeability is obtained for a mix made with 100% 12.5 mm long fibres at a fibre volume fraction of 1.0%, whereas the highest coefficient of water permeability is given by a mix made of 100% 50 mm long fibres at a fibre volume fraction of 1.5%. It may be noted that the mix made up of 100% 50 mm long fibres at fibre volume fraction of 1.5% has the water permeability even higher than plain concrete which may be as a result of fibre balling taking place during mixing.

In general, as the amount of short fibres in a particular mix is decreased or short fibres are partially or fully replaced by relatively long fibres, an increase in the water permeability is observed. Further, as already mentioned, for all the fibre mixes tested in this investigation, the lowest coefficient of water permeability is obtained for a mix made with 100% 12.5 mm long fibres at a fibre volume fraction of 1.0%, whereas the highest coefficient of water permeability is given by a mix made of 100% 50 mm long fibres at a fibre volume fraction of 1.5%.

It can be seen from the results of water permeability, compressive strength, and split tensile strength tests obtained in this investigation that no single fibre combination can be adjudged as the best combination for all these properties; this may be due to the reason that a large number of fibre combinations have been tested in this investigation. However, for most of the fibre combinations employed in this investigation, it can be concluded that materials achieve the best performance at a fibre volume fraction of 1.0% in all the tests conducted on the hardened concrete, that is, water permeability, compressive strength, and split tensile strength. It will be in the fitness of things to examine the results in more detail and to arrive at an acceptable fibre combination for all the tests conducted. It can be easily observed that the best performance in terms of split tensile strength is given by a mix containing 33% 12.5 mm + 33% 25 mm + 33% 50 mm long steel fibres at a volume fraction of 1.0%. The best fibre combination for water permeability is 100% 12.5 mm long fibres, whereas the mix made with 33% 12.5 mm + 33% 25 mm + 33% 50 mm long steel fibres gives the second best performance. Similarly, the mix containing 33% 12.5 mm + 33% 25 mm + 33% 50 mm long steel fibres gives the second best performance in terms of compressive strength also. Hence, it can be concluded without much loss of accuracy that, for water permeability, compressive strength, and split tensile strength, a fibre combination of 33% 12.5 mm + 33% 25 mm + 33% 50 mm long steel fibres can be adjudged as the best combination.

## 5. Conclusions

Properties of HySFRC containing different combinations of steel fibres of different lengths and plain concrete in hardened state have been investigated. Tests such as compressive strength, split tensile strength, and water permeability were conducted on hardened concrete after 7, 28, 90, and 120 days of curing. A total number of 40 mixes were considered and approximately 1440 specimens were tested in this investigation. For convenience, the results of various tests at the curing age of 28 days are presented and discussed briefly in this paper as the trends are more or less similar at other curing ages. In case of water permeability, a maximum decrease in coefficient of water permeability of the order of 79.21% for a mix with 100% 12.5 mm long steel fibres, at a fibre volume fraction of 1.0%, was observed followed by a mix made of 33% 12.5 mm + 33% 25 mm + 33% 50 mm long fibres. For compressive strength, maximum increase of the order of 29.54% over plain concrete was observed in case of mix containing 100% 25 mm long steel fibres at fibre volume fraction of 1.5%. Similarly, 46.07% increase in split tensile strength of HySFRC was observed with respect to plain concrete with a fibre mix ratio of 33% 12.5 mm + 33% 25 mm + 33% 50 mm long fibres at a fibre volume fraction of 1.0%. A careful examination of the results indicates that a fibre combination of 33% 12.5 mm + 33% 25 mm + 33% 50 mm long steel fibres can be taken as the most appropriate combination for water permeability, compressive strength, and split tensile strength of HySFRC.

## Figures and Tables

**Table 1:** Various HySFRC mixes.

*V*_*f*_ (%)	Fibre mix proportion by weight (%)		
	12.5 mm long fibres	25 mm long fibres	50 mm long fibres
0.5, 1.0, and 1.5	100	—	—
	75	25	—
	50	50	—
	25	75	—
	—	100	—
	75	—	25
	50	—	50
	25	—	75
	—	—	100
	—	75	25
	—	50	50
	—	25	75
	33	33	33

*V*_*f*_: fibre volume fraction.
